# A compact and realistic cerebral cortical layout derived from prewhitened resting-state fMRI time series: Cherniak's adjacency rule, size law, and metamodule grouping upheld

**DOI:** 10.3389/fnana.2012.00036

**Published:** 2012-09-06

**Authors:** Scott M. Lewis, Peka Christova, Trenton A. Jerde, Apostolos P. Georgopoulos

**Affiliations:** ^1^Brain Sciences Center, Veterans Affairs Health Care SystemMinneapolis, MN, USA; ^2^Department of Neurology, University of Minnesota Medical SchoolMinneapolis, MN, USA; ^3^Department of Neuroscience, University of Minnesota Medical SchoolMinneapolis, MN, USA; ^4^Department of Psychiatry, University of Minnesota Medical SchoolMinneapolis, MN, USA; ^5^Center for Cognitive Sciences, University of MinnesotaMinneapolis, MN, USA

**Keywords:** resting state fMRI, hierarchical tree modeling, brain networks

## Abstract

We used hierarchical tree clustering to derive a functional organizational chart of 52 human cortical areas (26 per hemisphere) from zero-lag correlations calculated between single-voxel, prewhitened, resting-state BOLD fMRI time series in 18 subjects. No special “resting-state networks” were identified. There were four major features in the resulting tree (dendrogram). First, there was a strong clustering of homotopic, left-right hemispheric areas. Second, cortical areas were concatenated in multiple, partially overlapping clusters. Third, the arrangement of the areas revealed a layout that closely resembled the actual layout of the cerebral cortex, namely an orderly progression from anterior to posterior. And fourth, the layout of the cortical areas in the tree conformed to principles of efficient, compact layout of components proposed by Cherniak. Since the tree was derived on the basis of the strength of neural correlations, these results document an orderly relation between functional interactions and layout, i.e., between structure and function.

## Introduction

Resting (i.e., task-free) fMRI is becoming an increasingly employed method by which to infer functional connectivity between brain areas. This body of research since the first paper by Biswal et al., published in 1995, has led to a multitude of presumed associations among various cortical areas and possible brain networks (Sporns, 2010). These results come from correlating time series of resting BOLD fMRI data after they are filtered down to very low frequencies (typically <0.1 Hz). These time series are typically nonstationary, i.e., they contain trends but also show autocorrelations for other reasons, commonly due to autoregressive (AR) and moving average (MA) processes. Commonly, all three components (trends, AR, and MA) are present in fMRI time series. Although these processes in resting fMRI time series may be of intrinsic value with regard to their biological origin, they will have to be removed prior to correlating the time series because, if left intact, the fundamental assumption of independence of errors (residuals) in the least square regression is violated and spurious correlations will be obtained. The removal of trends, AR, and MA dependencies can be done in various ways, including the Box-Jenkins AutoRegressive Integrated Moving Average (ARIMA) procedure (Box and Jenkins, 1970; Box et al., 2008), the linear transfer modeling approach (Liu, 1991; Pankratz, 1991), or a combination of linear or nonlinear detrending and fitting an ARMA model. The time series obtained as a result of eliminating these intrinsic processes are call “innovations,” to denote that they carry new information, unrelated to their history. Innovations are practically white noise (if the elimination process above is successful) and the overall procedure is called “prewhitening,” a term coined by Press and Tukey (1956, p. 220; see also Blackman and Tukey, 1959) to denote the fact that the spectrum of the innovations is white, i.e., prewhitened. (See the Appendix below for a brief discussion of the basic issues in time series analysis and their proper statistical treatment.) Locascio et al. (1997) drew attention to the pitfalls of correlating raw fMRI time series and outlined the correct procedures for computing valid correlations. Since 1995, we have applied prewhitening to fMRI data to detect task effects (Tagaris et al., 1995, 1997) and to perform brain network analyzes during cognitive processing (Georgopoulos et al., 2005, 2006; Lewis et al., 2006). In addition, we have used this method of correlating prewhitened neural time series of magnetoencephalographic (MEG) data to investigate task differences (Leuthold et al., 2005) and to assess the potential for such correlations to discriminate among, and classify, brain diseases in the resting state (Langheim et al., 2006; Georgopoulos et al., 2007, 2010; Engdahl et al., 2010).

In a recent paper, (Christova et al., 2011) we reported on the general results from a voxel-by-voxel correlation analysis of prewhitened resting fMRI BOLD time series. The main finding was that correlations are strongest within an area, followed by correlations between the same areas in the opposite hemispheres (homotopic areas), next between an area and other areas in the same hemisphere, and lastly between an area and areas in the opposite hemisphere (excluding the homotopic ones). These findings support the hypothesis that the strength of correlation between cortical areas reflects the density of overall anatomical connectivity, given the well-known strong local connectivity within an area, the strong interhemispheric connectivity between homotopic areas, the substantial, overall ipsilateral connectivity and the relatively sparse contralateral connectivity. Thus, we concluded that those results essentially follow the known pattern of the strength of anatomical connectivity, and that they could be inferred from this pattern under the reasonable assumption that there is an ongoing neural activity and communication among the various areas. In the present paper, we present the results of a more detailed analysis of our findings (Christova et al., 2011) obtained using a hierarchical tree modeling approach to reveal relations among specific areas. The layout of the cortical areas in the resulting tree (dendrogram) resembled closely the actual cortical layout of various areas and conformed to principles of efficient, compact layout of components proposed by Cherniak (Cherniak, 1994, 1995, 2012; Cherniak et al., 2004).

## Materials and methods

We analyzed a correlation matrix obtained from prewhitened resting fMRI BOLD series in 18 human subjects, as described in a previous paper (Christova et al., 2011). Details concerning subjects and methods are given in that paper and are summarized below.

### Subjects

Eighteen healthy human subjects participated in these experiments as paid volunteers. They ranged in age from 21 to 44 years; 9 were men (32.9 ± 2.2 years, mean ± SEM; range: 25–44 years) and 9 were women (25.2 ± 1.1 years; range 21–32 years). All subjects participated in the study after providing informed consent, in adherence to the Declaration of Helsinki. The study protocol was approved by the respective Institutional Review Boards.

### Task

The experimental task was simple, short, did not require a practice session and engaged the brain in a stable condition. Subjects lay supine within the scanner and fixated their eyes on a spot in front of them in the center of the screen. The absence of eye movement during this fixation period was verified by using an eye tracking system (ASL eye tracker, Applied Science Laboratories, Bedford, MA). Subjects were asked to remain still. Participants wore earplugs to reduce the scanner noise.

### Image acquisition

Blood oxygenation level dependent (BOLD) contrast functional images were acquired with a whole-body 3T MRI scanner (Magnetom Trio, Siemens, Erlangen, Germany) at the Center for Magnetic Resonance Research of the University of Minnesota using a gradient echo echo-planar imaging (EPI) (T2^*^) sequence with the following parameters: echo time (TE) = 23 ms; repetition time (TR) = 2 s; flip angle = 90°; in-plane resolution, 3 × 3 mm; slice thickness, 3 mm without inter slice gap. Whole-brain functional volumes (*N* = 203) of 38 axial slices covering the whole brain, cerebellum, and brain stem were obtained for each subject. A high-resolution anatomical T1-weighted 3D flash scan was obtained with the following parameters: *TE* = 4.7 ms; *TR* = 20 ms; flip angle = 22°; in-plane resolution = 1 × 1 mm; slice thickness = 1 mm; 176 slices in total.

### Data extraction

All analyzes were performed on the BOLD time series signal acquired per individual voxel in the whole brain of each subject. Coordinates in Talairach space for each voxel, as well as the BOLD intensity for each voxel, were extracted using Brain Voyager QX (v.1.10, Brain Innovation B.V., Maastricht, The Netherlands). Slice scan time correction was performed using *sinc* interpolation based on the information about TR and interleaved order of slice scanning. Three dimensional motion correction was performed to correct for small head movements, if present, by spatially aligning all volumes of a subject to the first volume using rigid body transformations. The estimated parameters of translation and rotation were inspected and did not exceed 3 mm or 2°. The 3-D volumes were then aligned with the corresponding 3-D anatomical volumes and normalized to standard Talairach space (Talairach and Tournoux, 1988). Matlab (R2008b, Mathworks, Natick, MA, USA) programs were implemented to enable BOLD time series extraction from the volume time course and anatomical mask available from Brain Voyager. For each subject, 203 functional images were acquired continuously, yielding a sequence of 203 BOLD signal values per voxel; of these, the first 3 volumes were discarded, leaving a time series of 200 BOLD values for analysis. Because the coefficient of variation is higher in the vicinity of large vessels and outside of the brain (Kim et al., 1994), we analyzed only voxels with coefficient of variation of no more than 5%.

A high-speed database server called Talairach Daemon (Lancaster et al., 1997, 2000) was used for automatic brain segmentation of individual brains in Talairach space. Talairach coordinates of each voxel were used to search the Talairach Daemon database (www.talairach.org, v.2.4.2) for the Talairach label using the “single point” search option. All voxels of the gray matter of the following 26 areas of the cerebral cortex in the left and right hemispheres were analyzed (for a total of 52 areas): correlations were obtained between the following areas of the left and right hemispheres: Precentral Gyrus, Superior Frontal Gyrus, Middle Frontal Gyrus, Inferior Frontal Gyrus, Paracentral Lobule, Medial Frontal Gyrus, Postcentral Gyrus, Superior Parietal Lobule, Inferior Parietal Lobule, Angular Gyrus, Supramarginal Gyrus, Precuneus, Superior Occipital Gyrus, Middle Occipital Gyrus, Inferior Occipital Gyrus, Cuneus, Lingual Gyrus, Superior Temporal Gyrus, Middle Temporal Gyrus, Inferior Temporal Gyrus, Transverse Temporal Gyrus, Fusiform Gyrus, Cingulate Gyrus, Anterior Cingulate, Posterior Cingulate, Parahippocampal Gyrus. The mean *x,y,z* coordinates of each area and its average volume (across the 18 subjects) are given in Table 1.

**Table 1:** **Mean *x-, y-* and *z*-Talairach coordinates and mean volumes (mm^3^) of 52 areas of the cerebral cortex of 18 subjects**.

**Cerebral area**	**Mean *x***	**Mean *y***	**Mean *z***	**Mean volume**
L_Precentral	−42.3	−7.2	39.4	10167.0
L_Superior_Frontal	−17.2	36.9	36.7	13167.0
L_Middle_Frontal	−36.0	25.9	30.8	14783.0
L_Inferior_Frontal	−44.5	21.7	3.2	8191.5
L_Paracentral	−6.2	−31.5	52.7	2550.0
L_Medial_Frontal	−6.7	26.6	27.1	10794.0
L_Postcentral	−40.3	−27.2	47.1	8248.5
L_Superior_Parietal	−26.0	−58.3	52.2	3244.5
L_Inferior_Parietal	−48.1	−41.0	40.3	7582.5
L_Angular	−43.9	−66.9	33.9	505.5
L_Supramarginal	−54.6	−46.6	30.2	1203.0
L_Precuneus	−13.0	−61.5	41.1	11618.0
L_Superior_Occipital	−35.6	−81.1	26.3	528.0
L_Middle_Occipital	−35.1	−83.3	6.1	2749.5
L_Inferior_Occipital	−33.6	−85.5	−8.2	1213.5
L_Cuneus	−11.1	−82.9	18.7	8146.5
L_Lingual	−12.4	−77.3	−3.2	4962.0
L_Superior_Temporal	−51.7	−17.4	1.6	10010.0
L_Middle_Temporal	−55.3	−36.3	−1.1	9009.0
L_Inferior_Temporal	−53.9	−37.5	−14.3	2535.0
L_Transverse_Temporal	−50.6	−20.9	11.5	1332.0
L_Fusiform	−39.9	−49.1	−15.6	5530.5
L_Cingulate	−6.9	−7.8	35.6	6937.5
L_Anterior_Cingulate	−6.0	33.2	8.8	4252.5
L_Posterior_Cingulate	−8.0	−54.5	15.3	2754.0
L_Parahippocampal	−23.9	−25.0	−11.7	5001.0
R_Precentral	44.1	−7.0	39.3	10749.0
R_Superior_Frontal	19.2	38.0	35.2	14063.0
R_Middle_Frontal	37.3	26.1	31.6	15624.0
R_Inferior_Frontal	46.7	22.0	4.9	8244.0
R_Paracentral	6.8	−31.4	53.1	2776.5
R_Medial_Frontal	7.5	26.6	26.7	10829.0
R_Postcentral	41.9	−27.0	47.4	8149.5
R_Superior_Parietal	27.5	−58.3	52.6	3481.5
R_Inferior_Parietal	49.0	−40.8	40.5	6873.0
R_Angular	43.9	−65.3	34.2	582.0
R_Supramarginal	52.7	−45.0	31.1	1231.5
R_Precuneus	13.4	−61.6	40.6	12014.0
R_Superior_Occipital	36.3	−81.0	26.6	388.5
R_Middle_Occipital	35.3	−83.0	6.5	2755.5
R_Inferior_Occipital	33.4	−85.2	−8.3	1143.0
R_Cuneus	11.8	−82.6	19.5	7707.0
R_Lingual	13.1	−76.3	−3.1	4957.5
R_Superior_Temporal	52.0	−14.1	−0.3	9825.0
R_Middle_Temporal	55.6	−33.0	−2.8	8463.0
R_Inferior_Temporal	53.7	−35.0	−15.8	2460.0
R_Transverse_Temporal	52.7	−19.9	11.5	1287.0
R_Fusiform	40.8	−48.6	−15.6	5145.0
R_Cingulate	7.9	−8.5	36.0	7749.0
R_Anterior_Cingulate	6.9	34.0	8.5	4234.5
R_Posterior_Cingulate	9.0	−54.4	15.0	2706.0
R_Parahippocampal	24.3	−25.6	−11.2	4452.0

### Data preprocessing: prewhitening the raw bold time series

Initial inspection of the BOLD time series from many voxels revealed that they were non-stationary with respect to the mean and highly autocorrelated. (The variance did not vary much along the series.) Since we were interested in calculating correlations between these time series, it is required, from first principles (Box and Jenkins, 1970; Box et al., 2008), that individual series be rendered stationary and nonautocorrelated for their correlation (i.e., not spurious). For that purpose we prewhitened each series using an ARIMA (15, 1, 1) model which yielded practically white noise innovations, i.e., stationary and nonautocorrelated residuals (see Christova et al., 2011 for details).

### Hierarchical clustering analysis

Next, we analyzed a correlation matrix obtained from the prewhitened resting fMRI BOLD series. We computed correlation coefficients between 52 cortical areas (see above), as follows. For a pair of areas, e.g., A and B, all voxel innovations time series in A were correlated with all voxel innovations time series in B. The correlation coefficients were *z*-transformed (Fisher, 1958; Christova et al., 2011), averaged, and the means converted back to correlations. Because correlations could take negative values, a constant (=2) was added to these means to transform them to a proximity measure. Finally, the data were averaged across subjects to obtain a 52 × 52 area proximity matrix which was subjected to a hierarchical tree modeling analysis. For that purpose we used the Hierarchical Cluster procedure of the IBM-SPSS statistical package (version 20) using the squared Euclidean distance as measure and the average between-groups linkage Unweighted Pair-Group Method using arithmetic Averages (UPGMA) as the clustering method. The result was visualized as a dendrogram in which the various cortical areas were segregated in various groups. Finally, standard statistical methods were used where needed (Snedecor and Cochran, 1989).

### Permutation testing of cherniak's compactness

We further analyzed the placement of cortical areas in the tree in the context of Cherniak's compact component placement theory (Cherniak, 1994, 1995, 2012; Cherniak et al., 2004). In general terms, this theory states that in a component-interacting system, an optimal strategy to optimize compactness is to place individual components as close to each other as possible, according to the strength of their interaction. Cherniak has posited three basic postulates of this theory, as follows. (1) The adjacency rule: “if components are interconnected, then they are positioned contiguously to each other, other things being equal” (Cherniak, 1995, p. 523); (2) the size law: “the larger the proportion of a total optimal system that the evaluated subsystem is, the better its optimization” (Cherniak, 2012, p. 365); and (3) the metamodule grouping: “if a set of connected components is optimally placed, then a set of metamodules, each consisting of a subset of those components in the same positions, is also optimally placed” (Cherniak et al., 2004, p. 1084). In our application, the component placement corresponds to the location of a brain area in the derived tree. We tested all three of the postulates above by a procedure comprising the following steps.

1. We computed *M* = 51 successive pairwise distances *D* (given a total of 52 areas) between brain areas in the derived tree:
  (1) $$D_{tree}^{i}={\left({\left(LR_{a}-LR_{b}\right)}^{2}+{\left(PA_{a}-PA_{b}\right)}^{2}+{\left(IS_{a}-IS_{b}\right)}^{\text{2}}\right)}^{1/2}\;$$
  where *i* is an index of successive distance (*i* = 1, *M*); *LR*, *PA*, *IS* are Talairach coordinates: Left-Right(negative→ positive), Posterior-Anterior (negative→positive), and Inferior-Superior (negative→positive); and *a*, *b* are successive brain areas in the tree.
2. We calculated the average distance
  (2) $${\bar{D}}_{tree}=\frac{\text{1}}{M}\sum_{i\;=\;1}^{M}D_{tree}^{i}$$
3. We randomly permuted *N* times the placement of these areas in the tree and recomputed the distances *D*^*k*^_*perm*_ as above (where *k* denotes a specific permutation, *k* = 1 to *N*) and their average ${\bar{D}}_{perm}^{k}$.
4. We compared each ${\bar{D}}_{perm}^{k}$ to ${\bar{D}}_{tree}$ and counted how many times ${\bar{D}}_{perm}^{k}<{\bar{D}}_{tree}$.
5. We computed the grand average
  (3) $${\bar{\bar{D}}}_{perm}=\;\frac{\text{1}}{N}\sum_{k\;=\;1}^{N}{\bar{D}}_{perm}^{k}$$
6. Finally, we computed the “compactness index” for the complete set as the ratio
  (4) $$c^{set}=\frac{{\bar{\bar{D}}}_{perm}}{{\bar{D}}_{tree}}$$
  as a measure of gain in compactness of area placement in the tree solution, as compared to the long-term average yielded by the permutations; the higher the *c*, the higher the gain in compactness. This procedure was applied to the tree derived as above from all 52 cortical areas of both hemispheres (after rectifying the LR coordinates) (*M* = 51), and, in addition, to trees derived separately for the left and right hemispheres. In the latter cases, *M* = 25, given 26 areas in a hemisphere.

The analysis above tested the presence of the adjacency principle on the whole set. The postulates of the size law, and metamodule grouping were tested by applying the same procedure to random subsets of areas in the tree, as follows. Let *L* be the size of a subset of the tree, i.e., the number of areas in a subset, *L* ≤ *M*. The measures ${\bar{D}}_{tree}^{subset}$, ${\bar{D}}_{perm}^{subset}$, $\;{\bar{\bar{D}}}_{perm}^{subset}$, *c*^*subset*^ were computed for each subset whose size *L* was varied systematically; the origin of a subset was randomly selected in each permutation from the full range of allowable tree locations of serial order 1 to *M-L*. Since *L* ≤ *M*, the comparison of *c*^*subset*^ to *c*^*set*^ would inform us whether the size law holds, which would predict that *c*^*subset*^ < *c*^*set*^. On the other hand, the frequency of occurrence of ${\bar{D}}_{perm}^{subset}<{\bar{D}}_{tree}^{subset}$ would inform us whether the metamodule grouping holds, which would predict that subsets of the tree would still be compact, as compared to those with random permutations, i.e., that the frequency of occurrence of the inequality above would be close or equal to zero. In this analysis, we used the following parameters: *N* = 1000 and 10 < *L* ≤ *M*.

## Results

**Figure 1:**
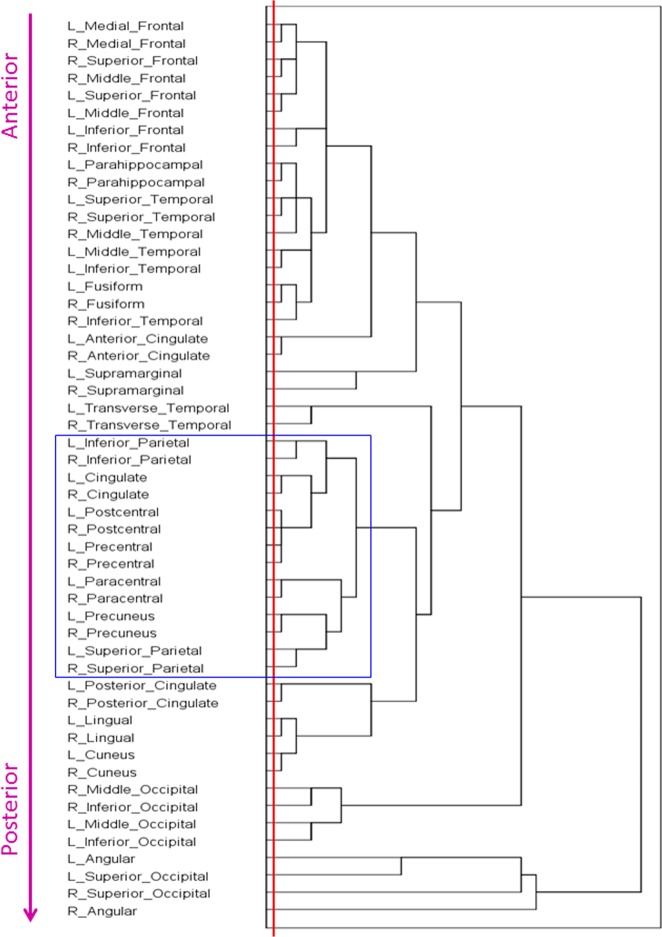
**Dendrogram derived by hierarchical tree clustering of 52 cortical areas (see text for details)**.

The dendrogram obtained is shown in Figure 1. The following can be seen. First, the tree consists of several partially overlapping clusters. Second, a section of the tree at the lowest level (red line) indicates the close clustering of interhemispheric (homotopic) areas. And third, there is a systematic progression of areas clustered, from anterior to posterior. We quantified this relation as follows. For each area, we calculated the average Talairach coordinates (Talairach and Tournoux, 1988) of all voxels and all subjects. Then we performed a multiple linear regression analysis between the serial order of the location of an area in the tree and its Left-Right (LR; negative→positive), Posterior-Anterior (PA; negative→positive), and Inferior-Superior (IS; negative→positive) coordinates, as follows:

(5) Tree order = a + bLR + cPA + dIS

Where *a-d* are regression coefficients. The equation obtained was:

(6) Tree order=11.13+0.013(LR)−0.345(PA)+0.213(IS)

**Figure 2:**
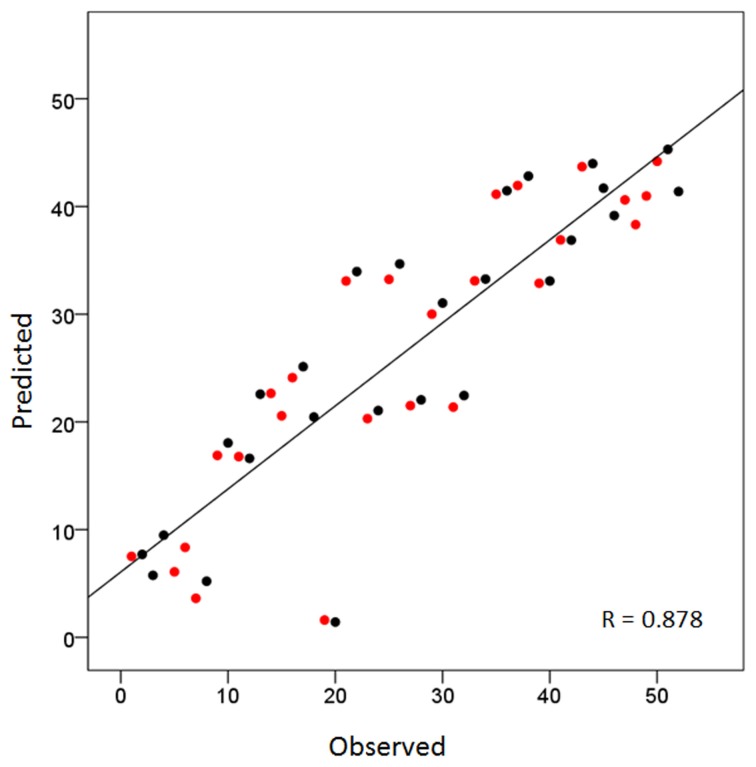
**Scatter plot of the sequence of cortical areas in the dendrogram of Figure 1 (from top to bottom) against the predicted sequence using the average Talairach coordinates (per area) as the independent variables (see text for details).** Red and black denote left and right hemispheres, respectively. Homotopic areas cluster closely as doublets of red and black circles.

**Figure 3:**
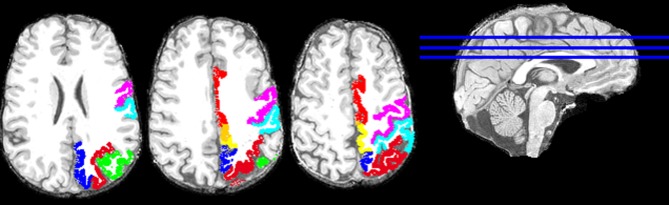
**Areas of the fronto-parietal cluster, enclosed in the blue box in Figure 1, are plotted on the T1-weighted structural images of one subject (left hemisphere).** Three axial slices are shown. Cortical areas of the left hemisphere are presented with the following colors: red, Cingulate Gyrus; violet, Precentral Gyrus; cyan, Postcentral Gyrus; yellow, Paracentral Lobule; green, left Inferior Parietal Lobule; blue, left Precuneus; and dark red, left Superior Parietal Lobule.

Overall, this was highly statistically significant (*F*_(3,48)_ = 53.8, *P* < 0.001) and with excellent fit (*R*^2^ = 0.771). As expected (since the homotopic areas clustered closely together), the LR coefficient was not statistically significant (*t*_51_ = 0.432, *P* = 0.668). The most significant was the PA coefficient (*t*_51_ = −12.4, *P* < 0.001), followed by the IS coefficient (*t*_51_ = 4.3, *P* < 0.001). Figure 2 illustrates these results by plotting the predicted tree order against the observed one. These results indicate an orderly progression in the tree from anterior and inferior to posterior and superior. A fronto-parietal cluster is contained within the blue box in Figure 1 and its component areas are shown in Figure 3.

### Permutation testing

#### Adjacency rule

The adjacency rule states that “If areas *a* and *b* are connected, then *a* and *b* are contiguous” (Cherniak, 1994, p. 2421); in the present case, we take “connected” to mean “interacting synchronously,” as quantified by the zero-lag correlation of the prewhitened resting fMRI time series. The adjacency rule can be tested by counting how many times the average consecutive distances between areas in the permuted tree would be smaller than the average distance obtained from the derived tree, i.e., ${\bar{D}}_{perm}^{k}<{\bar{D}}_{tree}$. We found the following with respect to the 52 areas and *N* = 1000 permutations, there was not a single case where ${\bar{D}}_{perm}^{k}<{\bar{D}}_{tree}$; this was true also for *N* = 1,000,000. The same results were obtained for the separate analyzes performed for each hemisphere. These findings demonstrate that the tree layout of cortical areas is much more compact than any random permutation would yield, up to *N* = 1,000,000.

#### Size law

**Figure 4:**
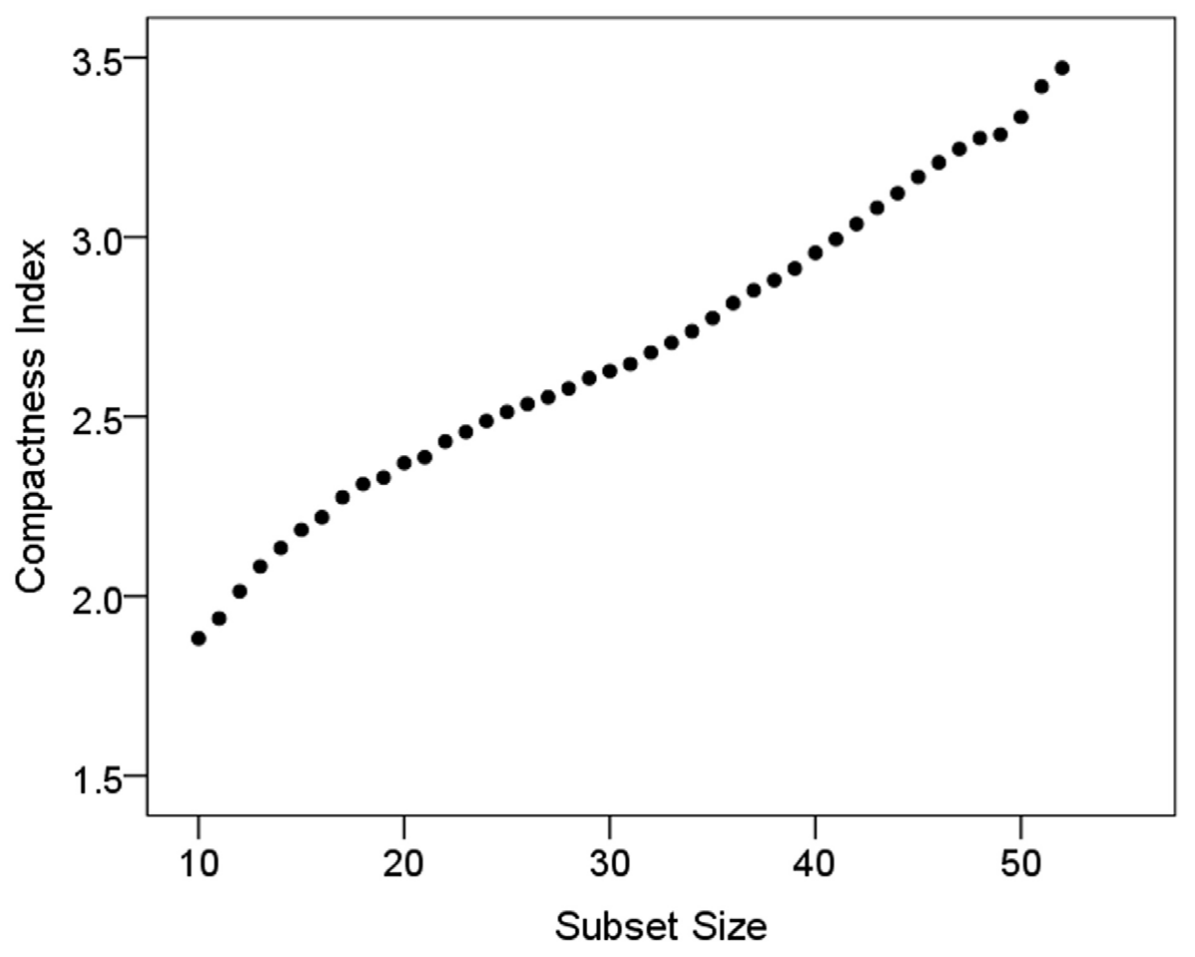
**Compactness index is plotted against subset size (see text for details)**.

The size law states that subsets of a compact layout would be less compact than the whole, i.e., that *c*^*subset*^ < *c*^*set*^. We evaluated this by computing those variables for subsets of *L* = 10, 11, 12,…, 51, 52 for the 52-area bilateral tree, and *L* = 10, 15, 20, 25 for the 26-area left and right hemisphere tree. For this analysis, 1000 subsets of each size above were evaluated using random starts along the tree (up to *M* − *L*, see “Materials and Methods”); the area placement in a subset was permuted 1000 times. This yielded 1000 values of *c*^*subset*^ per subset size *L*. Figure 4 plots the geometric mean of *c*^*subset*^ (average compactness index) against subset size. (Since *c*^*subset*^ is a ratio, it was log-transformed before averaging). It can be seen that, as predicted by the size law, *c*^*subset*^ is smaller for smaller subset sizes, and increases in a practically linear fashion as the subset size increases. A similar increase was observed for individual hemispheres.

#### Metamodule grouping

The metamodule grouping conjecture states that “If a set of connected components is optimally placed, then a set of metamodules, each consisting of a subset of those components in the same positions, is also optimally placed.” (Cherniak et al., 2004, p. 1084). We tested this conjecture by counting how many times the condition ${\bar{D}}_{perm}^{subset}<{\bar{D}}_{tree}^{subset}$ occurred, i.e., how many times the average distance between consecutive areas in a permuted subset of the tree would be smaller than the observed distance in the original sequence. We did not find any such instance in the testing of any subset of any size in the case of the 52-areas bilateral tree and in the case of the 26-areas left and right hemispheres. This finding shows that subsets of the tree layout are always more compactly placed than when randomly permuted, as predicted by the metamodule grouping conjecture.

## Discussion

### Methodological considerations

Our study differs fundamentally from all other studies of resting fMRI in four major respects. First, our analysis of functional connectivity was based on correlations computed between prewhitened resting fMRI time series, in contrast to other studies which have correlated time series without paying due consideration to their stationarity (i.e., constant mean over a long time; Bartlett, 1978) or the presence of AR (dependence on previous values) or MA (dependence on variation of previous values) processes, all of which would render the correlations thus obtained invalid, ranging from inaccurate at best and totally spurious at worst, depending on the strength of these intrinsic factors (see “Introduction”). Although ways and methods for eliminating within-series dependencies may be available in statistical packages aimed to analyze fMRI data, it is not commonly stated what use, if any, has been made of these methods, and with what result or effectiveness in achieving that goal. Serious concerns regarding this problem and the adequacy of various approaches implemented by existing fMRI analysis packages have been raised recently (Lenoski et al., 2008; Monti, 2011; Eklund et al., 2012).

In general, the treatment of this issue in published studies takes one of three forms: (1) In some studies, it is explicitly stated that filtered (to low frequencies) but non-prewhitened (i.e., detrended plus ARMA) time series have been used for correlations; in this case, the computed correlations are in doubt. (2) In other studies, the use of packages is mentioned (e.g., SPM, FSL, etc.) but without details as to what modeling on the time series was done and whether this was effective in eliminating dependencies; in this case, the computed correlations are in doubt too due to the lack of detailed information about this issue. (3) Finally, in other studies nothing is mentioned about this problem, so there is no way to tell what has been done, and, therefore these correlations are also in doubt. To ameliorate this uncertain situation, we propose that the Durbin-Watson statistic (Durbin and Watson, 1951) be reported together with every correlation analysis. This statistic evaluates the autocorrelation function of the residuals from the correlation analysis, and small values indicate spuriousness of the correlation (see also Draper and Smith, 1981, pp. 151–169). Alternatively, the Box-Ljung test on these residuals could be reported (Ljung and Box, 1978), which also evaluates the presence of significant autocorrelations. Both of these tests are readily given in standard statistical packages (e.g., IBM-SPSS). The advantage of this proposal is that no details of the kind of prewhitening procedure need to be given (e.g., nonlinear detrending, etc.): we only need to know whether the computed correlation is potentially spurious or not, and these statistics (with their associated statistical significance) will provide that information. We discussed this issue in detail, and illustrated results from our data, in a previous publication (Christova et al., 2011).

Second, our dataset comes from extensive, voxel-by-voxel correlations, specifically from more than a billion pairs of BOLD time series, instead of correlating averaged times courses from regions of interest (ROIs). No spatial smoothing was applied to the BOLD data, hence the analysis is uncontaminated from potentially artificial correlations imputed by the commonly applied substantial spatial smoothing. Although such smoothing is appropriate for certain analyzes (e.g., the detection of task effects in ROI-based analyzes) it is inappropriate for voxel-by-voxel correlations. To our knowledge, our correlation analysis (Christova et al., 2011) is the most extensive of its kind on record.

Third, our approach differs from others in that we analyzed the whole correlation matrix of 52 available cortical areas (26 per hemisphere), unlike other studies which have typically focused on certain “seed” areas. Although this seeding is valid, the results obtained are by necessity restricted to that particular seed, and a general cortical functional connectivity cannot be obtained by stitching together findings from separate seed studies to arrive at a global picture. In contrast, we aimed from the beginning at the whole picture.

Finally, we analyzed the data using a multivariate clustering approach (hierarchical tree clustering) which enabled us to identify some general principles of cortical interactions and layout as well as derive more specific clusters of cortical areas. As is the case for every multivariate analysis (e.g., factor analysis [FA], multidimensional scaling [MDS], etc.) hierarchical tree clustering does not yield a unique solution. For example, FA will yield different solutions based on the application of a factor rotation, and the kind of rotation; and MDS will yield different solutions depending on the choice of the level of measurement (e.g., metric vs. nonmetric). The choice of parameters in such multivariate analyzes typically comes from considerations of the data themselves and the objectives of the analysis; for example, Young et al. (1995) argued convincingly for the use of nonmetric MDS for analyzing cortical connectivity. In our analysis, there were two parameters involved. The first is the distance measure for which we used the squared Euclidean distance as the appropriate measure for continuous data. The second parameter concerns the method for combining clusters for which we used the average between-groups linkage (UPGMA, see “Materials and Methods”). This method was found by Milligan (1980) to be the top performer in recovering clusters (at 99.8% correct rate), among seven other methods he investigated in hierarchical cluster analysis. As a result, this is the standard method of choice, and the default method in the IBM-SPSS statistical package we used.

### Interhemispheric integration

We observed a pervasive close clustering of homotopic (left-right) areas (Figure 1, red line). This is in keeping with the well-known strong anatomical connectivity between homotopic areas and obviously underlies interhemispheric integration (Gazzaniga, 1970). Although this fact has usually being taken for granted, interhemispheric integration has not been a prominent feature of postulated resting state networks (van den Heuvel and Hulshoff Pol, 2010).

### Clustering of cortical areas

It can be seen in Figure 1 that cortical areas cluster in a concatenated manner. Although there is a general progression from anterior to posterior, there is also fine structure in the clustering pattern, reflecting, most probably, cooperative relations. As an example, consider the cluster highlighted by the blue box in Figure 1 and illustrated in Figure 3. It consists of several areas (always integrated across hemispheres) which have been involved in visuomotor coordination. The most closely integrated sub-cluster includes the pre- and post-central areas of both hemispheres. Also of interest is the cluster consisting of paracentral, precuneus, and superior parietal lobules, all areas involved in visuomotor coordination and anatomically interconnected (Ferraina et al., 1997; Marconi et al., 2001; Margulies et al., 2009). This cluster exemplifies the proximity arrangement of cortical areas from different lobes (e.g., frontal and parietal).

Overall clustering of cortical areas based on correlations (i.e., without focus on a specific seed) has been reported previously (Cordes et al., 2002; Salvador et al., 2005; van den Heuvel et al., 2008, 2009). However, we cannot discuss them, since these correlations were apparently computed from non-prewhitened fMRI time series (as far as we can tell from the section “Materials and Methods” of these papers), and since no information was given regarding the nonspuriousness of the computed correlations (e.g., by providing the Durbin-Watson statistic).

### Resting state networks

Our results indicate an orderly functional organization of cortical areas adhering to known anatomical facts and the results of modeling and optimization studies (Young, 1992; Cherniak, 1994, 1995, 2012; Young et al., 1995; Cherniak et al., 2004). No special roles or prominence or peculiarities were found regarding areas putatively involved in such networks. We cannot comment on the existing literature in that topic, given that published resting fMRI correlational studies come from correlating nonstationary and/or autocorrelated time series yielding inaccurate correlations. The net effect of this inaccurate analysis is that claims based on such studies, and postulated brain networks derived from them, cannot be evaluated. The reason that resting state networks inferred from such correlations may be found to be in accord with known anatomical connectivity stems from the fact that an existing, true relationship is part of the equation but its relative contribution is undetermined due to the presence of nonstationarity and other factors inducing autocorrelation in the individual series (e.g., AR and MA processes).

To illustrate this point, consider the following example. The wind is blowing from the north with various gusts, and the branches and leaves of two adjacent small trees move accordingly in a direction from north to south and with various intensities, depending on the moment's gust. A recording of their motion provides two time series that consist of trends in the trees' motions induced by the wind. Now, a high correlation between these time series is found but this does not mean that the trees are connected: the correlation is due to the common wind. An analysis of the trends in the individual series will reveal very similar trends in their motion (they will differ because the trees are not identical in their physical properties), and an inference could be made about the characteristics of the wind that produced them but not about the connectivity of the trees.

However, in another variant of this example, consider that the trees are indeed tied loosely by a rope (in a functional sense). The wind blows and the tree motions are recorded. In this case, the correlation between the two time series will reflect both the influence of the wind *and* the constraint of the connectivity of the trees by the rope. A correlation between the two series could not really provide accurate information either for the wind or for the strength of connectivity between the trees, simply because the motion recorded is the result of both factors. Also, a child could come by and shake one of the trees: this will produce an additional ripple in the time series of the motion of that tree, and so on for other perturbations. To find the truth for the “true” connectivity between the two trees, the influence of the wind and other factors should be removed first, and this is what a prewhitening of the series would accomplish: “clean” the series by removing the trend (and other dependencies), and only then correlating them to find out the presence and strength of their functional connectivity. (Conceptually, this is similar to multiple regression analysis where the influence of certain factors are “partialed out” or “regressed out” to find the correct relation between the dependent and specific independent variable.) Finally, all of these considerations apply to frequency-domain approaches as well (Blackman and Tukey, 1959; Jenkins and Watts, 1968; Granger, 1969; Priestley, 1981).

### Cortical layout

The global organization of brain areas obtained by the hierarchical tree clustering analysis (Figure 1) resembled very much the actual layout of the cortex. The correspondence of the order of areas in the tree to the orderly progressing Talairach coordinates from anterior to posterior, primarily, and from inferior to superior, secondarily, is remarkable (Figure 2). This result is in accord with results and outcomes of modeling and optimization studies (Young, 1992; Cherniak, 1994, 1995, 2012; Cherniak et al., 2004). More specifically, our results can be interpreted in the context of Cherniak's component placement idea (Cherniak, 1994, 1995, 2012; Cherniak et al., 2004), as follows. Cherniak has proposed that the cortical layout reflects an underlying cost minimization principle, namely the cost of axon wiring between cortical areas. Although plausible, this cannot be tested rigorously in the whole cortex due to the lack of quantitative neuroanatomical information about the density of connections between areas (in monkeys or humans), a point that has been repeatedly made clear (see, e.g., Young et al., 1995; Caspers et al., 2011, 2012). Older (e.g., Caminiti et al., 1985; Barbas and Pandya, 1989) and recent studies (e.g., Barbas et al., 2005; Dancause et al., 2007; Petrides and Pandya, 2007) with an emphasis on quantifying anatomical connectivity in the monkey are usually focused on a specific set of areas (e.g., prefrontal, premotor, parietal, etc.) and, in addition, they differ substantially in the underlying tracing methodology to allow pooling of results. Be that as it may, although Cherniak's suggestion about axonal wiring cost optimization cannot be tested thoroughly, his idea about a compact cortical layout is very interesting and can be tested outside (i.e., irrespective of) anatomical considerations. The point is that, although the concept of “layout” implies structural/spatial considerations, a specific layout can be derived based on data other than structural/anatomical ones. For example, sections in a library are defined based on the content of groups of books; floors or large spaces in thematic buildings (e.g., Ministry of Education, headquarters of a company, hospitals, etc.) are allocated to similar operations (e.g., inpatient/outpatient operations, personnel department, fiscal department, etc.) within which elements (individuals, small groups of people, etc.) are doing similar operations, etc. This typically holds for aggregates of units; e.g., various research laboratories are placed near each other in larger “research areas,” as opposed to administrative offices, which are placed in “administrative areas,” etc. The cerebral cortex reflects the outcome of long-time evolutionary pressures, and its layout seems to reflect overall spatial placements of areas conforming to the strength of their zero-lag correlations. It is very possible that this interaction is associated with anatomical connectivity, or with other aspects of neural communication as well. What is remarkable, however, is that this cortical layout was derived from purely functional considerations, since the Talairach coordinates of cortical areas did not enter in any of the calculations that led to the derivation of the tree.

### Conflict of interest statement

The authors declare that the research was conducted in the absence of any commercial or financial relationships that could be construed as a potential conflict of interest.

## Appendix

### On correlating time series

It is an undisputed fact that correlation (or regression) between any two time series is valid if, and only if, the series are stationary, i.e., if the mean of the series is constant throughout the time course and thus independent of the location of the time instant where it is calculated along the series (Priestley, 1981; Box et al., 2008; Jenkins and Watts, 1968; Bartlett, 1978). If this assumption does not hold, i.e., if the series are nonstationary, then spurious correlations are obtained. This issue had been recognized in the early twentieth century (Yule, 1926) but was brought forcefully to the foreground in the 1970s in the field of econometrics (Granger and Newbold, 1977). It ultimately led to a Nobel Prize awarded in 2003 to Clive Granger for (among other achievements) demonstrating that “the statistical methods used for stationary time series could yield wholly misleading results when applied to the analysis of nonstationary data.” (From Nobel Prize citation: http://nobelprize.org/nobel_prizes/economics/laureates/2003/press.html). Granger also discussed this issue in his Nobel Prize Lecture (Granger, 2004). The solution to the problem is to render the series stationary which can be achieved by differencing (i.e., taking successive differences in the series) or by the application of various detrending procedures. The hallmark of nonstationarity is the presence of autocorrelations in the series which can be detected by calculating the autocorrelation and partial autocorrelation functions (ACF and PACF, respectively). Suitable detrending will remove autocorrelations due to trends. However, a suitably detrended series may still show significant autocorrelations due to the presence of other processes, typically AR and MA processes (Box and Jenkins, 1970). The presence of an AR process indicates a dependence of a given value on previous values (irrespective of trend), whereas the presence of a MA process indicates a dependence of a given value on the variation (“random shock”) of previous values. Such dependencies need to be removed before correlation or regression analyzes are performed to ensure that the correlation obtained is truly due to the relation between the two time series and does not simply reflect, or is contaminated by, the time history of the series themselves. In a similar approach, as that applied for detrending, AR and MA dependencies are detected and removed. When this preprocessing is complete, i.e., when trends are removed and AR and/or MA dependencies eliminated, the resulting series are stationary and nonautocorrelated, which means that they are now ready to be correlated. Because there are no dependencies on previous values, the new “clean” series are called “innovations.” The standard way to accomplish this task consists of a three stage process (Box et al., 2008): (1) identify the sources of dependencies in the series (“model identification”), (2) calculate the coefficients for these dependencies (“model estimation”), and (3) take the residuals (innovations). As the final diagnostic check, the innovations time series should be stationary and non-autocorrelated. As mentioned above, there are three major sources of dependencies, namely trends (“Integrated” series), AR, and MA processes. The three stage process above used to identify, estimate and remove these dependencies is called ARIMA, from the initials (AR Integrated MA) (Box and Jenkins, 1970). Since the innovations series are essentially white noise, the data preprocessing above is called “prewhitening,” a term coined by John Tukey in 1956 (Press and Tukey, 1956) in the context of spectral analysis of time series. Detailed exposition, discussion and remedies for these problems can be found in any textbook on time series analysis (e.g., Priestley, 1981; Box et al., 2008), as detailed in a previous paper (Christova et al., 2011). It is unclear why this fundamental point has been so pervasively ignored in functional neuroimaging, while it has been commonplace in other fields, notably econometrics, for at least the past three decades. An obvious reason is that correlating autocorrelated time series requires special care of which the general neuroscientist is unaware. For example, a fundamental assumption of least squares linear regression is that the error terms be independent, and violation of this assumption invalidates statistical testing of the significance of regression slope, since its standard error is wrongly estimated. Although this is standard textbook knowledge (see, e.g., Snedecor and Cochran, 1989), it is not generally appreciated that regressing (or correlating) autocorrelated time series violates exactly this fundamental assumption, leading to inflated correlations (Pierce, 1979). In fact, in two early influential papers on fMRI data analysis, it was explicitly assumed that the errors in regression analysis between time series are independent, an erroneous assumption (Friston et al., 1995; Worsley and Friston, 1995). The detrimental effect of non-independent errors in regression analysis has been lucidly exposed by Box et al. (1978). It is interesting that the uncertainty as to how to correlate time series is clearly present in most recent textbooks of fMRI data analysis (Ashby, 2011; Poldrack et al., 2011).
